# Recruitment and Retention of Rural Health Professionals in Minnesota

**DOI:** 10.1111/1475-6773.14453

**Published:** 2025-02-22

**Authors:** Hannah MacDougall, Selam Woldegerima, Carrie Henning‐Smith, Teri Fritsma, Andrew P. J. Olson

**Affiliations:** ^1^ University of Minnesota School of Social Work Saint Paul Minnesota USA; ^2^ University of Minnesota Medical School, Medical Education Outcomes Center, Office of Medical Education Minneapolis Minnesota USA; ^3^ Division of Health Policy and Management University of Minnesota School of Public Health Minneapolis Minnesota USA; ^4^ Minnesota Department of Health Saint Paul Minnesota USA; ^5^ Division of Hospital Medicine, Department of Medicine University of Minnesota Medical School Minneapolis Minnesota USA; ^6^ Division of Pediatric Hospital Medicine, Department of Pediatrics University of Minnesota Minneapolis Minnesota USA

**Keywords:** medically underserved area, motivation, qualitative research, rural health, workforce

## Abstract

**Objective:**

To qualitatively explore the reasons health professionals decide to practice in rural areas.

**Study Setting and Design:**

Exploratory, cross‐sectional, semi‐structured qualitative interview and focus group study using thematic analysis with a convenience sample of health professionals in rural Minnesota. Interviews and focus groups were conducted virtually and in person, respectively, between August 2023 and March 2024.

**Data Sources and Analytic Sample:**

Primary interview and focus group data were collected from 19 individual interviews and 3 focus groups (*n* = 16) with health professionals in rural Minnesota. Interview and focus group recordings were transcribed, deductively coded, and analyzed using constant comparison.

**Principal Findings:**

Rural health professionals cited autonomy and breadth of practice and patient connection as rewarding and challenging components of practice that were distinctly rural. Barriers to recruitment and retention of rural health professionals included lack of housing (especially rental and short‐term) and accessible childcare. Potentially promising considerations when recruiting and retaining health professionals include loan forgiveness programs, the appeal of increasing racial and ethnic diversity in rural areas, and the ease of community health advocacy efforts.

**Conclusion:**

Our findings suggest that to recruit and retain rural health professionals, stakeholders could highlight autonomy and patient connection, reduce childcare and housing barriers, and explore community strengths such as racial/ethnic diversity and opportunities for advocacy.

**Summary**

- What is known about the topic?
- Currently, the vast majority of Health Professional Shortage Areas in the United States are in rural or partially rural areas.
- Past quantitative research found that factors such as growing up in a rural area, rural training program participation, and having autonomy at work were associated with rural health practice.
- What this study adds
  ○ A qualitative exploration of what drives health professionals to practice in rural areas.
  ○ Rural health professionals stated their autonomy and close patient connections were unique to rural practice and that lack of housing and childcare were barriers to rural practice.
  ○ Avenues to explore when recruiting health professionals include rural loan forgiveness programs, the appeal of racial/ethnic diversity in some rural communities, and opportunities to advocate for community health initiatives.

## 1 Introduction

Rural residents experience worse health, on average, compared to urban residents [1, 2, 3]. These health inequities can be partially attributed to barriers to care (e.g., long travel distances to access healthcare [4]) and diminishing access to facilities (e.g., those caused by hospital closures) [5, 6, 7]. In addition, there are substantial health workforce shortages in rural areas [8], which further exacerbates the barriers to care. While recent quantitative research has examined factors associated with rural practice [6, 9], qualitative research is needed to further understand the nuanced decision‐making that impacts where health professionals choose to practice. Such information is fundamental for informing future policy and health system efforts aimed at mitigating the rural healthcare workforce shortage.

Prior research has demonstrated that a multitude of factors influence healthcare professionals' decisions to work in rural areas, with the most salient and consistent factor being whether the professional grew up in a rural area [6, 9]. For example, Fritsma et al. found that growing up in a rural region is the most significant and constant predictor of working in a rural area across health occupations in Minnesota [9]. In the same study, the authors also identified that among advanced practice registered nurses (APRNs), physicians, physician assistants (PAs), and registered nurses (RNs) factors such as loan forgiveness, participation in a rural training program, having autonomy in practice, and broad scope of practice were associated with an increased likelihood of rural practice, independent of the effect of having grown up in a rural area [9]. Fritsma et al.'s uncovering of the salient factors associated with rural practice provided the framework used to dig deeper into these considerations throughout this manuscript.

Through interviews and focus groups, we sought to understand the nuance behind these factors to more fully grasp what, if anything, could help catalyze an increase in the rural health workforce. The aim of this study was to further explore the factors associated with the decision to live and work in rural areas among healthcare professionals in Minnesota to inform efforts aimed at recruiting and retaining rural health professionals.

## 2 Methods

### 2.1 Study Design

This qualitative study, employing a thematic analysis framework [10], was designed to expand on past quantitative findings with regard to motivators of rural health practice [9, 11]. Details of sample design, data collection, and analytic methods are summarized below.

### 2.2 Sampling

We used convenience sampling to recruit both individual interview respondents and focus group participants. We recruited licensed health professionals for individual interviews via emails sent to Minnesota health system leaders, and we contacted these same health system leaders regarding focus group participation. Health system leaders then distributed our recruitment email to licensed health professionals within their health systems, and these health professionals had the option to participate. Only those who were licensed health professionals (or in a training program for such a profession or an administrator with dual roles) in rural areas of Minnesota were included in the sample. Recruitment emails noted that individual interviewees would receive a $25 gift card and refreshments would be provided for focus group attendees. Respondents filled out a Qualtrics survey indicating their interest and availability. Individual interview respondents were then emailed an electronic consent form, which conveyed the objectives of the study. Focus group participants completed a demographic survey and a consent form prior to participation. This study was approved by and conducted in accordance with the University of Minnesota institutional review board.

### 2.3 Data Collection

Our research team consisted of PhD‐level researchers and graduate research assistants with expertise in rural health. We designed interview and focus group guides based on previous quantitative work [9], which identified key considerations associated with rural practice for Minnesota health professionals (Table 1).

**TABLE 1:** Distinctly rural components of practice illustrative quotes.

Theme	Illustrative quotes
Autonomy/breadth of practice	*I enjoy the autonomy of being able to be creative and being able to plan interventions so that they're functional for patients and most beneficial to them. Because I'm rural I get to use more of the full scope of my practice. Because if I were in a bigger city there would be, it would be more specialized. So, I mean, it just depends on what a person would prefer but I get to use all of the skills I learned and kind of keep up my education in all the areas*.
	*I think the benefit is having more autonomy and going deeper with patients with the time that I have with them and doing more well‐rounded stuff. So instead of just saying, well you're here for a sore throat, but also your blood pressure is high, and we should probably get you up to date on these vaccines and I think that's beneficial*.
	*Because I'm in a rural area now I have to be up to date in all of these areas and I can't specialize and just be for this one set population and be an absolute expert. I, like, need to be well versed in all of the areas and with adults, pediatrics, with the different diagnoses*.
	*But absolutely you want to bounce things off somebody. Maybe you want to go ask this person who, you know, has a little more education…You can call down [to a different health care system], but it's not really the same, you know, as crossing paths. And much like a kid, you know if you're playing outside, and you're excited you see a butterfly and you want to share it with your friend? You're also playing by yourself and there's no one to share it with. You know it works, it's just a little bit harder*.
Patient connection	*…you hear about, like, “everyone knows each other's business” or like seeing patients at times that you don't wanna be, like, on duty…I don't like to go in the stores. I tend to run into patients and it just kind of makes you, you never want to cross the boundary and, all the HIPAA (Health Insurance Portability and Accountability Act) laws, so I just kind, like, put little blinders on and yeah, go in here and leave*.
	*I do Walmart pick up, so I don't always want to talk to people in the grocery store because I went to [Redacted name of store] the other week and I ran into three patients. They started asking medical questions and, like, I'm literally here to pick out a fishing lure. So sometimes it's really overwhelming and not what you want to be doing. It's almost never what I want to be doing. Yeah, like, to the point where I just don't go to church anymore*.
	*One of the things that makes me stay is the difference between where I trained in the (urban setting) at multiple hospitals and here is the opportunity to really make an impact in the community through service to the people, you know, this is our community and it's our neighbors. It's our family and friends. It's very, I would say, intimate and at times that makes it really hard, but it makes it really rewarding to know that we are here as the healthcare entity to be there for them in their toughest times and I think there's just a certain level of ownership in that and pride in that*.
	*I'm a respiratory therapist and worked inpatient and outpatient and one of the most rewarding things is I had this patient come in the ER. He was frantic, he was short of breath, just you know how frantic they get, and I walked in the room, and he goes, “I know you!” and he instantly calmed down and just felt the sense of somebody knew who he was and some of his history, and I was there to help him. So, I think that is also some of the relationships that you develop in a rural community. Sometimes, unfortunately, it's your family members. But it's also patients that you develop a relationship with*.

### 2.4 Data Analysis

All interviews and focus groups were recorded and transcribed using Zoom. Transcripts were then uploaded to NVivo 14 qualitative software for analysis. We used a thematic analysis [10] and deductive coding approach and selected a coding frame based on previous work pertaining to survey data responses on rural health professional practice [9]. Specifically, we derived initial codes from past quantitative work, which identified the following factors as influential when choosing rural practice: growing up in a rural area, considerations about the specific rural area, family considerations, having a broad scope of practice, receipt of loan forgiveness, training and preparation, financial incentives, ability to specialize, and desire to work with certain patients [9]. We used a constant comparative method of coding and created subcodes such as “extended family lives in the specific rural area” and “spouse's career is in the specific rural area” within the “considerations about the specific rural area” code [12]. In the first coding stage, two researchers with expertise in the subject matter read each transcript multiple times and then separately assigned quotes to the existing coding frame. These two researchers then compared coding decisions, wrote analytic memos, and discussed emerging concepts and categories beyond the initial coding frame. In the second coding stage (axial coding), existing codes were merged into overarching themes [13]. We adhered to the Standards for Reporting Qualitative Research (SRQR) and the NICE qualitative checklist to ensure our methods and reporting were clear and robust (Table 2).

**TABLE 2:** Barriers to living rural illustrative quotes.

Theme	Illustrative quotes
Lack of childcare	*I think if the healthcare system were able to and could develop in‐house childcare we would have an opportunity to hire more, and not necessarily just licensed healthcare professionals, but also housekeepers, nurses' aides, you know some of those positions where they struggle to find someone to watch their kids on off hours, early mornings, you know, cause you have to be at work at 6:30 am. Well, most daycares don't open by 6:30 so I think that hinders our application pool when the health system doesn't have its own*.
	*Childcare is a huge issue. People choose to accept or not accept (a position) based on limited childcare options. And so, I think it is a huge challenge to draw people to the area. There's just not the amount of resources that you would see in a big city*.
	*Childcare is tough. I think I kind of lucked out on that one. I was very heavily pregnant and saw my current daycare lady with her daughter in a clinic visit, and she's like, are you looking for daycare, and I'm like, yes, yes I am…oh it almost fell into my lap, which I'm very fortunate about. We've had her for years now, but that is definitely a challenge out here*.
	*We have terrible childcare. We just don't have enough of it. So that definitely was not a draw. We really struggled to find childcare, and we've been through multiple different places…you know I have the means to be able to pay for some of the more, like, YMCA (Young Men's Christian Association) daycare that's just a little bit more of a group setting and things like that. But I would say in general, that's a real tough thing here*.
	*I was lucky that my husband stayed home when we came out here, and so I didn't really have to worry too much about childcare. But there's a tremendous shortage of childcare everywhere*.
	*Fortunately, my kids are at an age where I don't have childcare worries anymore and my husband just stays home…so that's helpful*.
Lack of certain types of housing	*I think the housing market is challenging. I think you have to know somebody who knows somebody. So, our house wasn't even on the market when we bought it. I think that's been a big challenge for people*.
	*I think housing is hard to come by, I think if you have money, you can buy housing. Our physicians have never had trouble finding housing in our communities, but I think our lower paid staff do, because it could be challenging*.
	*Within the last probably 5 years here in [community name] had a whole bunch of new apartment buildings built… that we desperately needed…I think it's really important. I know one of my friends that I used to work with in the area she was so frustrated that there was no… like she wanted to rent an apartment, like, a nice apartment but that wasn't available and she left because of that but there was nothing really available, but now I see what we have and it, like, she would have been able to stay if we had had that, you know*.
	*You can't really find an apartment to live in…I think I even heard, like, a medical student came up here and had to stay with family because you're on a waiting list and there's not many options*.

## 3 Results

We conducted 19 virtual, in‐depth semi‐structured interviews with professionals including physicians, hospital administrators, speech‐language pathologists, occupational therapists, chiropractors, PAs, RNs, and nurse practitioners. All respondents were licensed health professionals, or medical students working in rural Minnesota. Interviews ranged from 8 to 45 min in length, lasted a mean of 26 min, and took place between August 2023 and March 2024 (Table 3).

**TABLE 3:** *Promising* possibilities illustrative quotes.

Theme	Illustrative quotes
Loan forgiveness	*It really didn't impact where I was going to work. I really didn't even know about student loan repayment or those kinds of things. It was really not on my radar and then I paid off all my loans before I even learned about it*.
	*I'm not sure if that's a thing for nurses or just physicians? I kind of don't think it's for nurses ‘cause that doesn't sound familiar*.
	*One thing I'd like to add, though, is I did get multiple medicine scholarships during medical school (to subsidize rural medical education opportunities)…So I think from a university standpoint…there that might be an opportunity to encourage people in rural medicine*.
Diversity	*There's a lot of people that are moving up from the cities to have the enjoyment of the rural area but also just more diverse patients… Rural areas are not, I don't know, a monolith like sometimes people paint the picture*.
	*I wanted a place where I could definitely speak Spanish, and you know, work with a diverse community. But I also wanted to have an ability to engage with underserved medicine and, you know, effectively (name of town) is special in that regard. And yeah, communities of this size and nature are they're both special and they're not because they're pretty much like what America is*.
	*One of the things I really like about (name of town), especially compared to where I grew up, is the diversity we have. We have a fairly large, mostly Mexican population, but we also have a lot of people from Central American countries and then we have a really large Somali population here as well. And I really like that*.
Community health advocacy	*We have, I mean we know our local leaders. The mayor and the chief of police and… the county commissioner and school superintendent…I definitely think we have more say, an influence, than a lot of other places both here and in the community. You know I always held an example that I started a community garden 10 years ago and just it was so easy, like there were no roadblocks, they were just like yep you can do that. It just wasn't that, I don't know, bureaucratic? There are parts that are sure but for a lot of those like community health initiatives they might be smaller, you have more access to who can pull it off*.
	*I think I am someone with a unique role and ability with the relationships and continuity that we build with patients and the direct and more immediate nature that you would be willing to partner with organizations or community members is, I think, more responsive and more agile in a lot of ways*.

We also conducted three in‐person focus groups between August 2023 and May 2024 at two rural Minnesota health system sites, which lasted an average of 45 min. Focus group participants were licensed health professionals (or medical residents) practicing in rural Minnesota from a variety of professional backgrounds including medical students and those with dual administrative roles. The first focus group took place with physicians and advanced practice providers (NPs and PAs) (*n* = 4); the second was with a mix of health professionals including nurses, respiratory therapists, and radiology technicians (*n* = 4). The final group was composed of physicians, administrators, and medical students (*n* = 8). The majority of individual interview respondents (*n* = 19) identified as female (*n* = 11), white (*n* = 15), had children (*n* = 17), and were between the ages of 28 and 62. Of focus group participants (*n* = 16), the majority identified as female (*n* = 12), white (*n* = 13), had children (*n* = 11) and were between the ages of 25 and 60 years.

### 3.1 Results of Thematic Analysis

#### 3.1.1 Distinctly Rural Components of Practice

Two themes emerged as neither wholly positive nor negative but rather unique to rural healthcare practice (Autonomy and Breath of Practice and Patient Connection), and we therefore coded them as “Distinctly Rural Components of Practice.”

### 3.2 Autonomy and Breadth of Practice

When asked what rural healthcare professionals enjoyed about rural practice, autonomy and breadth of practice were most frequently cited. While distinct concepts, respondents spoke about them in tandem, and they are therefore coded as one theme. This conflation was often discussed as a benefit where health professionals use their full scope of practice. In addition, several health professionals noted that patient health may benefit from physicians providing a breadth of services, and therefore more holistic care.

While many of the rural health professionals interviewed expressed gratitude for the variety in their working days, patient populations, and services provided, many professionals (primarily physicians) also noted that this variability can come with challenges. While breadth of practice can be beneficial, respondents noted that providing a wide variety of services can be overwhelming, especially when health professionals feel out of their depth in certain practice areas. Moreover, the same autonomy lauded by many health professionals also presented a challenge, as interviewees noted that being autonomous was sometimes lonely and overwhelming. Without colleagues to consult or celebrate with, health professionals often felt isolated.

Autonomy and breadth of practice were distinctly rural themes that were salient across interviews. Respondents noted that they had a high degree of independence and were expected to provide care for patients across the lifespan with a wide variety of medical needs. These components of rural practice were at times mentioned as attractive and beneficial and at other times, isolating and overwhelming.

### 3.3 Connection to Patients

The rural health professionals we interviewed almost unanimously noted that connection to their patients was the most rewarding part of rural practice. While the connection to patients was cited as a benefit, it was simultaneously acknowledged that these same connections were, at times, the most challenging part of rural practice. Because rural health professionals, especially those working in the communities with the smallest populations, were likely to encounter and know their patients outside of work, respondents reported that work‐life boundaries were often blurred.

Several rural health professionals commented on the impact of seeing patients while they were “off duty” and the challenges this could present. One focus group participant who works as a physician noted that [they] had significantly changed the way [they] participate in the community including avoiding physically going into grocery stores and no longer attending church. In addition to changing daily routines, some rural health professionals made other decisions to ensure they are afforded some anonymity in their home communities. Several focus group members noted that a mental health professional in their health system had intentionally moved to a different community than the one in which they worked to afford some separation from work/patients.

Despite these challenges, rural health professionals also spoke about the connection they feel with their patients as one of the main reasons they find rural health practice particularly meaningful. One interview respondent who works as a nurse stated [they] cried at the deathbed of a patient [they] had known for over a decade. Another physician stated that knowing patients are [their] friends and neighbors makes [their] work feel more rewarding in the community. In addition to feeling the intrinsic reward of caring for your friends and neighbors, several health professionals noted that this type of connected, longitudinal care led to better health outcomes for patients. A respiratory therapist we interviewed reflected on a moment when this was abundantly clear, when [their] presence calmed a patient so much, they were able to breathe again.

Having autonomy and breadth of practice in addition to close connections to patients were salient themes throughout the interviews and focus groups and were largely identified as positive qualities in rural practice. That said, respondents also shed light on the double‐edged nature of these themes, demonstrating that what is most appealing about rural practice can also be the most challenging. While these concepts may be motivators or deterrents to rural practice, respondents found these themes to be distinctly rural and therefore important to consider when attempting to recruit and retain future health professionals.

#### 3.3.1 Barriers to Living in Rural Areas

Respondents cited two primary barriers to recruiting and retaining health professionals in rural areas: lack of childcare and lack of short‐term/rental housing.

### 3.4 Lack of Childcare

Many respondents noted that lack of access to childcare in rural areas is a pervasive issue while adding that they believed high costs and lack of access to childcare were also urban and national issues. While all individual interviewees were asked whether they believed childcare was an issue in their community, the majority of interviewees who responded affirmatively skewed female and younger. We also found that concern about childcare spanned professional backgrounds and income. However, respondents noted that health system employees in varying income brackets likely experienced different childcare‐related barriers. For example, one physician noted that lower‐paid staff in the health system, such as housekeepers and nursing assistants, had irregular schedules with off‐hours work, creating an additional barrier to finding childcare.

In addition to health system employees having trouble finding childcare that met their scheduling needs, several respondents also noted that childcare availability affected the recruitment of health professionals. One PA noted that it is hard to draw professionals from urban areas to their area because there was no childcare available. Those professionals who did have childcare were quick to point out that they were “lucky” and were very clearly aware that their situation was not experienced by many in the community. Another PA shared that she “lucked” into childcare because one of her patients was a childcare provider. One physician noted their good fortune in finding childcare and stated that even with the privilege of a higher income, it had been a challenge to find. Several health professionals noted that they were fortunate to have a partner who was at home with children.

### 3.5 Lack of Certain Types of Housing

When discussing barriers to recruitment of health professionals, housing was frequently cited as an issue. Depending on the community, certain types of housing were particularly hard to find, with rentals, apartments, and smaller‐family homes being the most frequently mentioned. Indeed, one PA noted that without “insider knowledge” it was very challenging to purchase a home.

In addition to houses being sold quickly, several respondents stated that whether housing was an issue depended on which health professionals you were attempting to attract, noting that physicians/higher‐paid professionals had fewer issues finding housing than professionals in the middle‐ or lower‐income bracket positions. Lack of rental housing was a frequently cited concern for recruiting and retaining health professionals. One respondent noted that a colleague had left due to a rental shortage, but that [they] were heartened to see new apartments being built.

Despite new housing being built in one community, several professionals shared that they struggled to find housing for medical residents who lived in the community temporarily. This concern was particularly galling for those present in one focus group as they also expressed their desire to attract more medical residents to ensure rural educational pathway programs were available. The lack of housing and childcare available to rural health professionals was almost unanimously cited as barriers to recruitment and retention.

#### 3.5.1 Promising Possibilities

Respondents discussed three avenues for recruitment and retention that they felt were underexplored, including loan forgiveness programs for rural health professionals, advertising the racial and ethnic diversity in rural communities, and the possibility of community health advocacy. We coded these three themes as “promising possibilities” for rural health professional recruitment and retention.

### 3.6 Loan Forgiveness

Respondents stated that they were either unaware of rural health professional loan forgiveness programs in Minnesota or that they found these programs confusing. Loan forgiveness was only noted as a factor in considering rural practice for three interviewees and the rest of the individual interviewees either did not participate in loan forgiveness or relied solely on the federal Public Service Loan Forgiveness Program.

When discussing whether loan repayment was a motivator, across professions, these programs did not play a significant role. Indeed, a speech‐language pathologist noted that [they] did not realize there was a program until after [they] had paid off [their] loans. Further, four individual interviewees noted confusion about the possible programs and were unclear about eligibility for the MN‐specific loan forgiveness programs. They shared confusion about whether they were eligible based on where they lived, their professional background, and their subspecialties.

While loan forgiveness appeared unrelated to the choice to practice rural, several focus group participants noted that loan forgiveness or rural‐specific scholarships can play a helpful role in recruiting rural health professionals by subsidizing rural‐specific training programs. Despite the current lack of importance assigned to rural‐specific loan forgiveness programs, many of the respondents believed rural‐specific funding would be helpful if there was greater clarity on how to receive this support.

### 3.7 Diversity

While respondents noted that rural areas are often perceived as lacking in racial and ethnic diversity, several respondents voiced their desire to challenge this narrative. One physician noted that [they] decided to practice in a rural area because [they] wanted to work in “underserved medicine” and work with Spanish‐speaking persons in a diverse, rural community. [They] also noted that his community was both unique and common, and wanted to further challenge the belief that rural areas are a monolith. Another interview respondent had similar thoughts on how much [they] appreciated the racial and ethnic diversity in their rural community.

Interview and focus group respondents provided insight into the variation in racial and ethnic composition of rural areas and expressed an interest in challenging the myth that all rural areas are homogenous.

### 3.8 Community Health Advocacy

The final theme that emerged from our interviews and focus groups was that many rural health professionals felt they had a significant impact on their communities and could advocate for community health. A physician in one focus group noted that through community connections, [they] were astounded by how easy it was to create a community garden in the town. The lack of bureaucratic red tape and the connection to local leaders enabled this physician to lead a project that she found rewarding and beneficial to the health and vitality of the community.

In addition, another physician noted the possibility of partnering with community‐based organizations to advocate for patients and create community‐wide change. This physician expressed the benefit of rurality in terms of understanding the needs of patients and their families and being able to quickly and effectively partner with community‐based organizations to address community health issues.

Respondents shed light on three themes they felt were underutilized as recruitment and retention tools for rural health practice: loan forgiveness programs, the appeal of racial and ethnic diversity in some rural areas, and the ease of community health advocacy.

## 4 Discussion

Findings from interviews and focus groups reveal opportunities to address the persistent rural health professional workforce shortage issues in Minnesota. Specifically, respondents provided information on appealing aspects of rural practice, barriers that should be addressed, and ideas that may improve the experience, and thus sustainability, of rural health practice.

Autonomy was perceived as both an advantage and a challenge in rural health practice. The rural health professionals interviewed for this study often were caring for a large number of patients, and sometimes felt isolated, leading to a feeling of burnout. In addition, health professionals in rural communities often felt a blurring of lines between their professional and personal lives, with both positive and less positive effects. This heightened sense of responsibility, while empowering, also meant that providers were on their own for critical decision‐making, which was both mentally and physically taxing. However, interviewees did express that they had an increased ability to make decisions independently in rural practice, a fact corroborated by past research [14], and this was appealing to many interviewees. In sum, the individuals interviewed wanted to maximize their own autonomy while having appropriate support to provide high‐quality care. To address concerns of professional isolation and blurred boundaries, rural health systems could provide opportunities for learning and connection through virtual or in‐person gatherings where professionals could informally connect and/or learn strategies to hold boundaries and increase connection. Health professions schools may also play a role in facilitating these forums.

Similarly, the scope of practice in rural settings was described as broad by many participants, allowing them to use and apply their diverse set of skills. A broad scope of practice for rural health professionals has been documented in the literature [15], and our study found this could be rewarding, as it enabled healthcare providers to engage in a wide variety of medical procedures and treatments that they might not have performed in more specialized urban settings. However, this advantage came with the disadvantage of increased pressure on providers to manage complex cases that they might otherwise refer to specialist providers if such resources were readily available [16]. The lack of access to specialists meant that rural healthcare providers often extended their practice beyond their typical scope, which could be daunting. To address concerns regarding access to specialty providers and to provide consultation services, rural health systems could consider expanding their use of telehealth services to provide consultation and lessen feelings of isolation. One available resource is Project ECHO (Extension for Community Healthcare Outcomes), which uses video conferencing to provide training and advice to support primary care providers and increase access to specialty treatment, especially in rural and underserved areas [17].

Community connection could be emphasized as a valuable tool for recruiting healthcare professionals to rural areas. However, it is equally important for rural health systems to address the unique challenges that come with these close‐knit community interactions. As described previously, rural healthcare providers often encountered patients outside of work. These interactions raised questions such as whether to acknowledge patients in social settings, how to handle the treatment of patients they know personally, and how to respond to medical inquiries outside of work hours.

Addressing childcare and housing issues presents a valuable and actionable opportunity. If properly funded, initiatives to increase the availability of accessible, affordable childcare and housing (specifically rental housing) could substantially improve the recruitment and retention of healthcare providers in underserved areas [18, 19]. The Farm Bill, which is currently in negotiations in Congress, may include support for expanding childcare in rural areas [20]. However, it last passed in 2018 and is currently under a temporary extension, expiring December 31, 2024, and has yet to reach an agreement [20, 21]. While childcare remains a nationwide crisis regarding cost and availability, its effects are keenly felt by rural areas attempting to address the healthcare workforce shortage. Further, the challenges with accessibility and affordability of childcare may disproportionately affect healthcare professionals with lower remuneration, thereby exacerbating disparities. Federal and state support, such as tax credits for childcare, can play an important role in addressing the affordability of childcare; however, health systems and communities may need to do more to ensure the availability of childcare in rural communities.

Similarly, housing has long been the target of federal, state, and local policy, including funding for rental and home loan assistance, support for new construction, and zoning laws, but rural housing requires additional attention. Addressing the availability, affordability, and quality of rural housing—all of which are significant issues across rural areas [22, 23, 24]—is essential to addressing rural healthcare workforce shortages [25]. Health systems could consider directly investing in housing and/or converting their land assets into sites for temporary or permanent housing options. While rural residents are more likely than urban residents to own their homes [26], rural housing policy should also include a focus on rental stock, particularly for trainees and other temporary workforce.

Loan forgiveness programs are designed to alleviate the financial burden of student debts for rural health professionals [27]. It was notable that many participants were unaware of the state‐level loan forgiveness incentives when they decided to work in rural areas, making it a welcome bonus for them rather than a reason they chose to move to a rural area. This indicates a significant gap in the advertisement, publicity, and awareness of these programs, which, if addressed, could attract and retain more healthcare workers in underserved regions. Additionally, collaboration with state health departments, health systems, and educational programs could help reach a wider pool of healthcare workers who may not be aware of these programs. Lastly, several participants noted that they had access to health system‐level loan forgiveness programs, which could be another avenue for recruitment to rural areas.

Our interview and focus group respondents pushed back on the notion that rural areas lack racial and ethnic diversity; indeed, several respondents noted that the racial and ethnic diversity in their communities was exactly what brought them to the area or kept them practicing there. While is it important for health professionals to be aware that some respondents acknowledged experiencing racism in their communities, advertising the diversity of the community may be a draw to licensed health professionals, who may specifically seek out caregiving experiences with diverse communities. As rural areas across the US. become more racially and ethnically diverse [28, 29], this may become even more attractive.

A potentially enticing component of rural healthcare practice is the ability to affect local change with fewer bureaucratic hurdles than one might encounter in urban areas. Rural civic engagement and volunteerism varies by community [30, 31, 32], but offers an opportunity to affect local change and promote community health. This unique facet of rural life could be more widely promoted as a distinguishing and satisfying component of health practice in less‐populated areas. Moreover, rural health professionals, alongside their health system employers, could advocate for the very social programs that may aid in the recruitment and retention of professionals such as affordable short‐term housing and accessible childcare. To further address community public health, rural health professionals may also find advocating for existing funding mechanisms, such as value‐based payments, to be a meaningful way to improve the quality of care [33] and potentially address social drivers of health. Health systems can bolster recruitment efforts by emphasizing rural health professionals' potential ability to directly promote community health in rural areas.

## 5 Limitations

One of the main limitations of our study is its lack of generalizability. The study sample comes from one state and does not include individuals from all professions, which restricts the applicability of our findings to a broader population. While our research provides valuable insights into the specific groups studied, it does not account for potential differences across various professions. This oversight means that professional context and the unique challenges faced by different occupational groups were not considered in our analysis. Another potential limitation is that our sample relied on health system leaders distributing recruitment emails to their employees, which could lead to health professionals feeling pressured to participate. We attempted to mitigate this concern by providing a direct link for health professionals to use to sign up for interviews and health system leaders did not have access to this information. Future studies should aim to include a more diverse range of professions to enhance the generalizability of the results. Additionally, investigating differences across professions could provide a deeper understanding of how occupational factors influence rural practice.

## 6 Conclusion

There is an urgent need to address rural health workforce shortages, and these qualitative findings provide in‐depth, nuanced ideas generated by those most intimately connected to the issue. While autonomy may be a selling point to attract new providers, to retain them, health systems may need to address the flip side of autonomy exposed through these interviews: isolation and overwhelm. While promoting community connection is helpful, the findings suggest that it is also important for rural systems to provide clear guidance on how to handle community connections when boundaries feel blurred. Investments in short‐term and rental housing as well as childcare provided by health systems and other key stakeholders may aid in recruitment and retention efforts. Lastly, individual communities may wish to increase awareness surrounding rural‐specific loan forgiveness programs, the racial and ethnic diversity of their communities, and the possibility of public health advocacy and action for local health professionals. To ensure the ongoing vitality of rural areas, addressing the rural health workforce is vital and this study provides specific and actionable steps to promote this goal.

## Conflicts of Interest

Dr. Hannah MacDougall served on an advisory board at the Lown Institute for which she received an honorarium. Dr. Carrie Henning‐Smith serves as a board member for CentraCare. The other authors declare no conflicts of interest.

## Data Availability

Research data are not shared. All qualitative data gathered from interviews/focus groups are required to remain confidential as a part of the IRB process to ensure anonymity of interviewees is protected.
